# Nutritional Composition, Bioactive Compounds, and Volatiles Profile Characterization of Two Edible Undervalued Plants: *Portulaca oleracea* L. and *Porophyllum ruderale* (Jacq.) Cass

**DOI:** 10.3390/plants11030377

**Published:** 2022-01-29

**Authors:** Tamara Fukalova Fukalova, María Dolores García-Martínez, María Dolores Raigón

**Affiliations:** 1 Laboratorio de Fitoquímica y Productos Biológicos, Facultad de Ciencias Químicas, Universidad Central del Ecuador, Quito 170521, Ecuador; tfukalova@uce.edu.ec; 2 Instituto de Conservación y Mejora de la Agrobiodiversidad Valenciana, Universitat Politècnica de València, 46022 Valencia, Spain; magarma8@qim.upv.es

**Keywords:** healthy food, quality characteristics, nutritional composition, bioactive compounds, volatiles profile, undervalued plants

## Abstract

Wild edible plants are an important source of healthy food and have played an important role in traditional Mediterranean diets. In this paper, quality characteristics were typified in *Portulaca oleracea* L. and *Porophyllum ruderale* (Jacq.) Cass, undervalued plants inherent to the spring-summer season in the Valencian coastal region. Nutritional composition and bioactive compounds were analyzed and compared between plants in wild and organic cultivation conditions. Proximate analysis was carried out according to Association of Official Analytical Chemists methods. Total antioxidants were measured as 2.2-diphenyl-1-picrylhydrazyl hydrate and total polyphenols content via the Folin–Ciocalteu procedure. The HS-SPME technique was used to characterize the volatiles profile, and the polyphenol profile was evaluated by HPLC. The most important microelement was iron. Total antioxidants ranged from 4392.16 to 7315.00 μmol Trolox·equivalents 100 g^−1^ fw, and total phenolic content ranged from 99.09 to 391.18 mg gallic acid equivalents·100 g^−1^ fw. Results show that the content of antioxidants and phenols was higher in wild species than in cultivated ones. The volatiles profile revealed that *P. ruderale* was rich in monoterpenoids (48.65–55.82%), and fatty alcohols were characteristic in *P. oleracea* species (16.21–54.18%). The results suggest that both plants could be healthy foods and could have new sustainable agro-ecological potential for the local commercial sector.

## 1. Introduction

The Food and Agriculture Organization of the United Nations estimates that 75% of the genetic diversity of the world’s crops has been lost. Of the 7000 species that have been used as food, fiber, textile, medicine throughout history, only about 150 are cultivated for human and animal consumption [1]. The rest of plant species are underused and undervalued, causing loss of agrobiodiversity in territories [2,3], even though many of these plants have high economic, ecological, and food potential. Factors such as climate change, deforestation, and cultural erosion influence the disappearance of many plant species that at times were very important in human intake as healthy food, giving them the status of undervalued, with serious consequences for agriculture, nutrition, and food security [4]. In a context of climate, environmental, and social crisis, undervalued plant species are considered important to farms and farmers [5].

Providing the growing world population with healthy food based on sustainable and alternative food systems is a pressing social challenge of the 21st century [6], even more so considering the situation generated by the COVID pandemic, which essentially highlighted the need for change in many aspects of modern life, among which include food sustainability and the conservation of undervalued plants as future resources [7,8].

Today, part of this ethnobotanical heritage is being recovered, providing technical scientific information on the species composition, botanical value, and potential for current and future uses [9,10]. Originally, one of the identifying characteristics of the traditional Mediterranean diet was the introduction of the closest edible resources, which allowed for the incorporation of wild plants for consumption, some of which are still valid in current elaborations, forming part of the local dishes and enriching them with flavor and nutritional value. The gathering of wild edible plants is linked to their seasonality and is part of the traditional regional knowledge. Being seasonal plants, they play a fundamental role in response to climate changes due to their long process of natural selection [11,12]. 

Many plants have been revaluated and have received considerable attention, mainly focusing on ethnobotanical and pharmacological aspects. The potential of edible plants in terms of their nutritional and bioactive benefits have been investigated only in a few cases, despite representing a particular aspect of local biodiversity and being an important food source, especially in the Mediterranean Basin [13,14,15]. In this region, the environment is characterized by a greater abundance of endemic flora. The richness and diversity of wild species, their collection times, and edaphoclimatic and growing environments make it difficult to standardize their nutritional composition, causing heterogeneity of their components [16,17,18,19]. Consequently, the highest contents of bioactive components are obtained by respecting their temporality and optimal vegetative development [10].

Mediterranean traditions have made it possible for a considerable number of wild plants to remain present in the human diet [20]. These plants are still consumed locally, alone or in combination with cultivated species, in various ways, such as fresh (salad), cooked (soup and boiled), and as condiments for their organoleptic properties. The seasonal consumption of undervalued species allows, on the one hand, to minimize the resources used for their growth, since they easily adapt to environmental conditions, and on the other hand, offers a range of foods with seasonal alternatives throughout the year, providing a variety of meals and the development of a sustainable cuisine [21,22]. Consuming these plants prevents chronic degenerative diseases, cardiovascular diseases, and obesity, among others [23,24]. For this reason, traditional food items resort to the use of edible plants that are indistinguishable from medicinal plants, both of which form part of the biocultural diversity and regional culinary traditions [25]. The increasing demand for healthy foods is renewing interest in the use and research of undervalued wild edible plants.

Currently, conventional crops have displaced many of the once known and appreciated wild species, making them undervalued. Among these undervalued species are *Portulaca* *oleracea* and *Porophyllum ruderale*, both inherent to the spring-summer season in Mediterranean conditions. According to a previous ethnobotanical review, these selected plants have cultural relevance and are deeply rooted in the traditional cuisine of the Valencian coast [26,27,28,29,30,31].

Although they are edible wild species and are accessible owing to their abundance during their season, their nutritional and bioactive composition, as well as their phenolic and volatile profiles, have not been scientifically reinforced. Given this situation, we have proposed the following hypothesis: the undervalued edible species *P. ruderale* and *P. oleracea* provide variability in their nutritional, aromatic, and bioactive compounds depending on the environment’s growing conditions. For this purpose, the proximate analysis and quantification of bioactive components, in addition to other chemical constituents and the organoleptic matrix, were carried out. An analysis of the volatiles profile, which contains high-value functional components, was performed by headspace solid phase microextraction (HS-SPME) and gas chromatography mass spectrometry (GC/MS), and an analysis of the polyphenol profile was performed by HPLC. This work aims to be a reference to promote the inclusion of these plants as a nutritional alternative given the high demand for a balanced and healthy diet, and at the same time, due to their wide presence in the corresponding season, to diversify the intake of food and promote the use of traditional gastronomy, thereby establishing a sustainable path for potential new crops. This work also evaluated the differences between the composition of the two species in wild conditions and in organic farming conditions.

## 2. Results

The proximate nutritional compositions of fresh leaves and small tender stems of *P*. *ruderale* and *P*. *oleracea* were evaluated; this also included the most representative macrominerals and microminerals. In addition, the bioactive constituents were characterized under wild and organic farming conditions. Each sample consisted of approximately 1.5 kg of fresh plant with random collection. The data obtained are presented in Table 1. The results are reported as the mean of replicates, alongside the coefficients of variability (CV) of each value and *p*-value, which test the statistical significance of the estimated effect of environment growing conditions.

### 2.1. Proximate Composition

*P. oleracea* was the species with higher leaf moisture, which oscillated between 83.12% and 88.39%; in the case of *P. ruderale*, it ranged between 76.64% and 84.70%. In both species, the moisture content of the fresh parts increased under the wild growing conditions with significant differences, and variability of this parameter was low in all cases (between 0.03−1.31%). The dry matter content in the plants tested ranged from 11.61 g·100 g^−1^ to 23.36 g·100 g^−1^; the values of this parameter were significantly different between the cultivated and wild conditions for *P. ruderale* (*p* = 0.0000) and *P. oleracea* (*p* = 0.0140). The ash level was lower in species grown in wild conditions compared to their counterparts grown in cultivated conditions, and this difference was accentuated in *P. ruderale* (*p* = 0.0020). The amount of ash was high in *P. oleracea* with 2.62% (wild) and 3.39% (cultivated). Foliar crude protein concentration was found to be higher in wild growth conditions (1.89%) in the case of *P. ruderale*, presenting significant differences from its counterpart in organic cultivation conditions (*p* = 0.0002). For *P. oleracea,* the same patterns were observed without differences between wild and cultivated conditions. Fat accumulation in the leaves of the species studied was very low and its distribution was uneven across growing systems. For *P. ruderale*, the highest concentrations, with significant differences (*p* = 0.0156), were found in wild cultivation conditions (0.66%). For *P. oleracea,* the fat in the plants from the cultivated environment (0.99%) was three times higher than that of its counterpart in wild conditions. No difference in fiber levels were observed between the species studied in the two conditions, and they ranged from 2.39 to 5.50%. Carbohydrate levels were higher in *P. ruderale*, with a maximum in the cultivated species (17.50%). The same trends occurred in *P. oleracea*, with a higher level of this parameter reached in the cultivated species (8.41%). The significant differences were more pronounced in *P ruderale* (*p* = 0.0008). Energy values ranged from 28.0 to 78.75 kcal·100 g^−1^ fresh weight, with higher levels in cultivated plants compared to wild plants.

The coefficient of variety (CV) showed a wide discrepancy in the nutritional parameters such as crude fiber and carbohydrates, with 34.18% and 51.32%, respectively, in wild *P. ruderale,* demonstrating the excessive dispersion. In contrast, *P. oleracea* had the highest variability for nutritional parameters such as crude fiber (34.23%) and carbohydrates (10.22%) in cultivated species. The remaining parameters were less variable, with coefficients of variability oscillating between 0.03% and 5%. The nutritional parameters that showed the least variability value were protein (0.05%) and fat (0.10%).

### 2.2. Mineral Composition

The mineral composition of studied plants was measured and is recorded in Table 1. The most abundant macromineral in the edible parts was potassium in wild conditions for *P. ruderale* (515.28 mg·100 g^−1^ fw) and for *P. oleracea* (776.67 mg·100 g^−1^ fw), with no significant difference between growing conditions (*p* > 0.05) for *P. ruderale* and a significant difference for *P. oleracea* (*p* = 0.0000). Other abundant macrominerals were calcium and magnesium, which showed significant differences between growth environments, especially in *P. oleracea* (*p* = 0.0005 and *p* = 0.0000, respectively). The most prominent micromineral was Fe, with the highest content (1.80 mg·100 g^−1^ fw) in species from wild conditions for both plants, although there were no significant differences between growth conditions. The greatest mineral variability occurred for *P. ruderale* in calcium (27.29%) and potassium (9.55%) content when in wild conditions and sodium (19.49%) in cultivated conditions. In *P. oleracea*, calcium (15.19%) and potassium (22.08%) contents were the least stable for wild species, together with copper (7.14%).

The comparison of the nutritional composition as a function of the growth environment of each species was carried out considering statistically significant effects (*p*-value), which are indicated in Table 1 with the letters as a super index for each parameter analyzed, except for the caloric value obtained by calculation. The nutritional parameters with *p* = 0.0000 are the ones that differ the most depending on the growing environment.

### 2.3. Non-Nutritional Compounds

The non-nutritional compounds (considered by some authors as bioactive components [32]) were quantified and are presented in Table 1. These were antioxidants, total phenolic content, and chlorophylls (a, b, and total). The amount of total antioxidants in the fresh wild samples of both plants stood out, ranging from 4645.53 to 7315.0 (µmol TE·100 g^−1^ fw), with significant differences from the cultivated species. On the contrary, the total polyphenol content in *P. ruderale* was higher, although there was no significant difference between wild and cultivated species; the difference was significant in *P. oleracea* (*p* = 0.0021). However, the total phenols values presented the greatest variability among all the parameters studied, especially in wild *P. ruderale* (36.17%), followed by cultivated *P. oleracea* (35.83%). Chlorophyll content (a, b, and total) in fresh plant samples were higher in wild plants for both species, although significant differences between growing conditions were only present in *P. ruderale*, where the wild species was double the cultivated species for these parameters.

Similarly, Table 1 shows the significance value (*p*-value) for each of the bioactive compounds analyzed in the edible parts of the studied species, indicating the difference in their levels between wild and cultivated species, with the letters as a super index.

### 2.4. Polyphenols Profilere

In total, ten polyphenolic compounds from two categories—hydroxycinnamic acids and flavonoids—were identified and are listed in Table 2. Five phenolic compounds were detected in both species under the two growing conditions. They were hydroxycinnamic acids: chlorogenic, caffeic and *p*-coumaric acids; and flavonoids: quercetin and kaempferol. Three components were detected in *P. ruderale* and corresponded to gallic acid, rutin, and luteolin. The most abundant phenolic compound was chlorogenic acid in *P. ruderale* for both growing conditions without a significant difference between them, followed by *p*-coumaric acid with a higher concentration in cultivated organic conditions.

Gallic acid was not detected in *P. oleracea* and other phenolic compounds were higher in cultivated than in wild conditions with significant differences. For flavonoids, six different compounds were identified in *P. ruderale* and four *in P. oleracea,* without rutin or luteolin. Quercetin and myricetin as major compounds as a major compounds *P. ruderale* were only detected in its cultivated species. Myricetin in *P. oleracea* was higher, being significantly increased in organic cultivation conditions, and apigenin was detected only in the cultivated species.

Organic production systems were favorable for the synthesis of flavonoids in the two undervalued species. They also favored the synthesis of polyphenolic compounds in *P. oleracea*, but only *p*-coumaric acid synthesis in *P. ruderale.*

### 2.5. Other Chemicals

**Figure 1:**
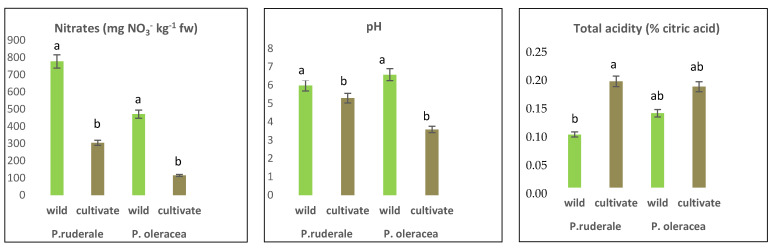
Nitrate concentrations, pH, and total acidity in fresh edible parts of *P. ruderale* and *P. oleracea*. The significant differences are visualized in letters. The letters a, b showed that difference exist and ab showed that difference not exist.

Other chemical components determined were nitrates and parameters related to the acidification of edible leaves (pH and total acidity); the are results presented in Figure 1. In both species, the nitrate concentration in fresh plant samples was higher in wild species than in cultivated species, with statistically significant differences (*p* < 0.05), showing a higher level in *P. ruderale* (777.3 mg NO_3_^-^ kg^−1^ fw) compared to *P. oleracea* (471.0 mg NO_3_^-^ kg^−1^ fw). Similarly, the pH value was significantly higher in wild plants compared to cultivated plants, and its highest value was recorded in *P. olerace* (6.6). Being inversely proportional, the titratable acidity showed opposite behavior in terms of pH, in which the cultivated samples presented higher values than the wild ones in terms of acidity. Total acidity did not show a significant difference between growing conditions in the case of *P. oleracea* (range 0.14–0.19%), while there was a significant difference in the case of *P. ruderale* (range 0.10–0.20%).

### 2.6. Correlations between Quality Parameters

The study of the correlations between nutritional, mineral, and bioactive compounds in the two plants studied showed different degrees of correlation between them. These correlations are presented for *P. ruderale* in Table 3 and for *P. oleracea* in Table 4. Since total chlorophyll is the sum of chlorophylls a and b, only its correlation is presented.

Among the nutrients of *P. ruderale*, crude protein has the highest number of correlations, with nine strong and significant relationships. Five of these relationships corresponded to a negative correlation with carbohydrates and minerals, and four to a positive correlation with fat, other minerals, and bioactive compounds. A strong positive relationship between crude protein and total chlorophyll was established (r = 0.990), which shows the strong relationship between the two parameters of absorbed nitrogen for this species. In contrast, crude protein from *P. oleracea* did not show any significantly strong relationship. Among the nutrients of *P. oleracea,* fat showed the highest number of correlations, also with nine strong relationships. Six of these relationships corresponded to a negative correlation with mainly minerals and bioactive compounds. The most complete negative relationship was observed between fat and sodium (r = −0.999).

Among minerals, copper showed the greatest number of strong relationships, while potassium and sodium showed no strong and significant relations in *P. ruderale*. In the case of *P. oleracea*, all the minerals showed a highly significant relationship except for Zn. Copper was the micromineral that presented the highest number of strong relationships in both species, such as the total antioxidants of the bioactive components. In general, the strongest negative relationships were observed in *P. ruderale*, while in *P. oleracea* positive relationships of similar magnitude prevailed.

### 2.7. Volatiles Profile Analysis

**Figure 2:**
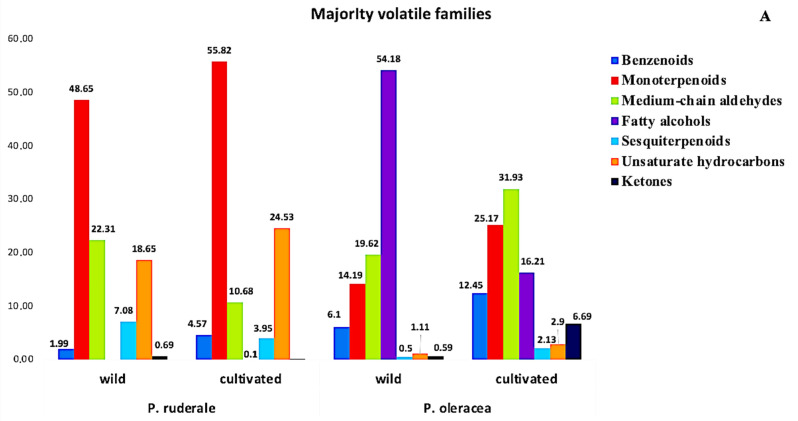

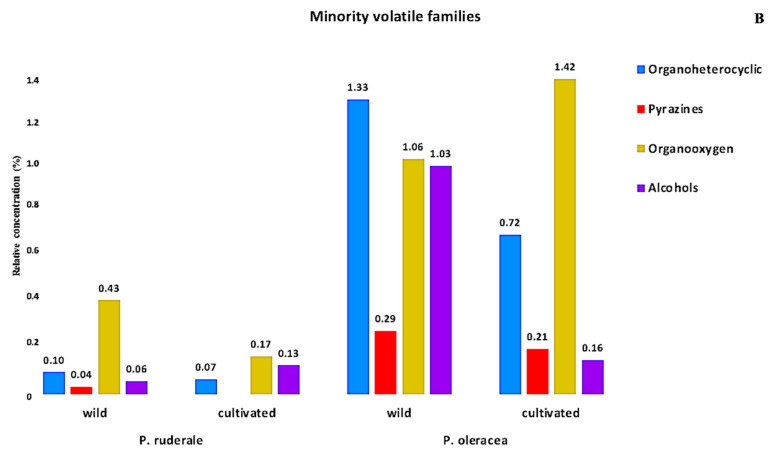
Relative content of the chemical families of volatiles in fresh leaves: (**A**) majority families of volatiles in wild and cultivated species of *P. ruderale* and *P. oleracea*; (**B**) minority families of volatiles in wild and organic cultivated species of *P. ruderale* and *P. oleracea*.

The volatile profile analysis revealed the presence of 11 chemical families. Figure 2 presents the relative percentage of each chemical family of volatile components detected in the fresh aerial parts of the plants studied. The majority of volatile families were: benzenoids, monoterpenoids, medium-chain aldehydes, fatty alcohols, sesquiterpenoids, unsaturated hydrocarbons, and ketones (Figure 2A). The minority volatile families were: organoheterocyclic compounds, pyrazines, organooxygen compounds, and alcohols (Figure 2B). The volatiles profile revealed that *P. ruderale* is rich in monoterpenoids (48.65−55.82%), and fatty alcohols are characteristic in *P. oleracea* species (16.21–54.18%).

Monoterpenoids were present in both species, although they predominated in cultivated *P. ruderale* (55.82%), more than in its wild counterpart (48.65%). Unsaturated hydrocarbons were the second family present in both species and stood out in organic cultivated *P. ruderale* (24.53%). Another family present in both species and growing environments was medium-chain aldehydes, which was greatest in cultivated *P. oleracea* (31.93%). Concerning benzenoid content, *P. oleracea* stood out in both growing conditions: wild (6.1%) and organic farming (12.45%). The highest amount of sesquiterpenoids was found in wild *P. ruderale* (7.08%), and cultivated *P. oleracea* stood out in ketones (6.69%). The family of fatty alcohols was characteristics only in *P. oleracea*, tripling the amount in wild conditions (54.18%) compared to cultivated conditions (12.45%). The families of smaller quantities are preponderant in *P. oleracea*, unlike *P. ruderale*, in both growing conditions. The organoheterocyclic family predominated, especially in the cultivated plants (1.33%).

The next families were organo-oxygens (1.42%) and alcohols, which emphasized were most prevalent in wild *P. oleracea* (1.03%). The pyrazine family was more pronounced in *P. oleracea,* where it reached higher amounts in the wild species (0.29%).

## 3. Discussion

As there is little demand for them and a lack of knowledge of their uses, some edible leaf species are undervalued and underused, which contributes to the loss of local agrobiodiversity. The two plants in this study are abundant in the Mediterranean spring-summer season. *P. ruderale* is an introduced species and *P. oleracea* an indigenous species. The characterization of nutritional, mineral, and bioactive constituents was carried out with the edible aerial parts of both plants. A bibliographic analysis in reference to these two species showed that their medicinal properties had been studied; on the contrary, there is little information about the nutritional quality and functional properties of these food plants, especially in the case of *P. ruderale*. Characterizing the quantitative properties (amount of nutrients) and qualitative properties (bioactive compounds present) could make an important contribution to the daily requirements of a balanced intake.

The most prominent nutritional parameters characterized were ash (total minerals), protein, fiber, and carbohydrates. Mostly, the nutritional levels of edible plants are related to their lifespan, which in turn depends on the species itself and humidity, temperature, and other environmental factors. The moisture content of the fresh samples of wild species was higher than that of the cultivated species, thus presenting a lower level of dry matter. In the research by Arias-Rico et al. [33], the average values of 85.3% for moisture in wild Mexican *P. ruderale* were described. The same species in this study presented similar water content in the same environmental conditions.

On the contrary, the extensively cultivated *P. ruderale* from Mexico, studied by Lara et al. [34], presented moisture values around 91.0%, which are probably due to the applied irrigation. On the other hand, the species studied, *P. oleracea*, presented a lower water content than that found by [35], with a value of 92.9%, and that found by [36], with a value of 91.23% in a wild environment. The diversity of environmental and geoclimatic conditions may be the cause of the difference in physicochemical properties, among which is moisture. The results obtained in this study are similar to those of [37], carried out in leaves of Greek *P. oleracea* from Mediterranean conditions, with 88.16% moisture.

However, the result obtained is within the normal moisture content for green leafy vegetables and shows a very stable foliar parameter for both species. High water content tends to decrease the energy density of food, making it important in obesity diets, and should be considered a true nutrient that ought to be part of the diet [38]. The higher water content in wild species may be due to the greater sponginess of the soil and the greater retention of water available to the plant in these wild conditions.

In this study, the highest total minerals content was found in *P. oleracea*, both wild and cultivated. The results obtained were higher than those observed in [35] in research with 1.22 g·100 g^−1^ on the same wild species from Brazil. The obtained ash results of our study are in concordance with [37], which found 2.40% ash in Greek *P.oleracea*. On the contrary, the ash in *P. ruderale* was higher compared to that obtained by [34], with 0.9 g·100 g^−1^ fresh weight in cultivated Mexican species, and lower than that obtained by [33], with 2.04 g·100 g^−1^ in wild Mexican species. This may be due to the growing conditions and edaphoclimatic characteristics of the respective geographical areas, especially in the case of *P. ruderale,* which is a plant native to South America. As ash is an index of the total mineral content in food, its levels in plants suggest the considerable availability of these constituents, which are considered essential for humans.

In the analysis of the protein obtained in this study, the wild species of *P. ruderale* showed a value very close to that found by [34] in *P. ruderale*, with a result of 1.8 g·100 g^−1^ fw; a lower content of this nutrient was found in its cultivated counterpart. Low content was found in *P. oleracea* in this study compared to hat reported by [35], with 2.03 g·100 g^−1^ fw. Conclusively, both species studied demonstrated low protein content when compared to some conventional fresh species such as parsley (3.0 g·100 g^−1^ fw), basil (14.4 g·100 g^−1^ fw), or mint (3.8 g·100 g^−1^ fw) according to [39]. As these are products of conventional agriculture, conditions of high nitrogen fertilizer content may be the cause of these differences.

The total lipid content in Mediterranean *P. oleracea* reported by [39] was a value of 6.90 g·100 g^−1^ db. The Greek *P. oleracea* had 0.23 g·100 g^−1^ fw of total fat according to the study carried out by [36], and our results corroborate that this species is an abundant source of vegetable fat. The same study highlights that fat is rich in omega family fatty acids, which are beneficial for human health. If compared to the values reported by [35], which are 0.36 g·100 g^−1^ fw, our *P. oleracea* exceeded the fat content, especially in cultivated conditions. When comparing the results reported by [34] in fat content for *P. ruderale* (0.3 g·100 g^−1^) on the fresh basis, it can be mentioned that the results obtained in this study from the fresh parts of cultivated species were superior, and even more so in wild species.

While the wild species of *P. ruderale* excelled in crude fiber content, surpassing the values (2.17 g·100 g^−1^ dm) of Mediterranean *P. oleracea* reported by [40] and the content of Swiss chard (1.0 g·100 g^−1^) by even more according to [39], the cultivated counterpart of *P. ruderale* and *P. oleracea* showed a lower level of crude fiber. Although the dietary fiber values found in this study are not high compared with the levels in parsley (7.26 g·100 g^−1^), fiber intake is essential to improving the digestion of food and in the prevention of certain diseases such as diabetes, atherosclerosis, obesity, and constipation [41]. However, due to the crude fiber values found in this study, both species are an attractive source of this nutrient. It should be added that factors such as the degree of maturity and the botanical variety together with different culinary processes can modify the fiber intake, as well as the other nutrients. It would be useful to compare the nutritional parameters of the fresh plants studied with those cooked in various ways such as stewed or boiled.

The highest calculated energy value was recorded in cultivated *P. Ruderale*, placing it above all the samples studied, although when compared with the Greek *P.* *oleracea*, it was lower (61.3 kcal·100 g^−1^ fw). This caloric level is due to the greater amount of carbohydrates and fiber in this plant; however, it corresponds to a moderate caloric level [38]. The study carried out by [42] highlights that the highest nutrient density/energy density ratios determine the nutritional quality index that can be useful in food selection, as it is scientifically proven that diets with lower energy density can help maintain a healthy weight and improve nutritional quality. Digestible carbohydrates are an additional source of energy in diets. However, a low-carbohydrate diet can reduce the risk of cardiovascular diseases [43], so *P. ruderale* and *P. oleracea* may be beneficial in this regard when their leaves and stems are consumed as fresh vegetables.

In relation to minerals, the levels of calcium, potassium, and magnesium stand out as macrominerals; and iron and zinc as microminerals, while sodium levels are minimal due to the ability of plants cell to keep sodium low in response to environmental conditions [44]. This predisposition makes vegetables a good choice for low-sodium diets. In *P. ruderale*, calcium is abundant, especially in its cultivated species, whereas in wild *P. oleracea,* the potassium content stands out. In both species, the amount of magnesium is also high. This may be due to geochemical conditions, such as soil composition and hydric state, among other factors. The most important route for the uptake of macro and microminerals in plants is through the roots; however, it has been observed that other tissues can also absorb minerals. The size of the metal ions plays a predominant role in this process. The ability to accumulate minerals is not a common characteristic of plants; rather, it is an evolutionary response. In addition, magnesium is present as a constituent of the chlorophyll molecules, from which it is released by gastric and intestinal secretions when the leaves are consumed. Its consumption is necessary to maintaining a healthy body since, together with zinc, it participates in many biological processes. Moreover, both minerals are essential in eye health. Complementarily, zinc is an essential trace element and must be provided on a regular basis as part of a healthy diet; it participates in the modulation of the immune system, is involved in various cell metabolism functions, and plays an important role in maintaining the concentration of tocopherols in plants [45]. Both species of *P. oleracea* are projected as good sources of zinc, similar to spinach, which contains 0.5 mg·100 g^−1^. The iron found in the study is uniform in cultivated and wild species. Iron is an essential microelement in food. According to [39], two conventional vegetables, watercress and spinach, are important sources of this element and with values of 1.30 and 2.27 mg·100 g^−1^ fw, respectively, and the plants studied can compete with these. Furthermore, the iron content in the plants in this research was similar to the value found in Argentinian *P. oleracea* (1.99 mg·100 g^−1^ fw) [46] and was surpassed by that of Brazilian *P. oleracea* (10.5 mg 100 g^−1^ fw) [36], both in wild growing conditions. Although our *P. oleracea* appears to be a species with promising levels of Fe and Zn, and *P. ruderale* with Fe and Ca, the interaction of these minerals with the crude fiber and total polyphenols present, should be considered since these factors negatively influence mineral bioavailability and may require more detailed studies. On the contrary, the micromineral copper positively intervenes in iron absorption. A contribution of microminerals such as Fe, Cu, and Zn is necessary for the biosynthesis of antioxidant enzymes that participate in the body’s oxide-reduction reactions [37,47]. In general, wild greens contribute more to the dietary intake of minerals than wild fruits [48].

The DPPH assay is a preliminary test to study the antioxidant effect of the plants. In the research carried out by [49] on *P. oleracea* leaves grown in Egypt, antioxidant activity was determined by the DPPH method with 147.78 (µmol TE·100 g^−1^ dm). This result contrasts with those obtained in this study for *P. oleracea,* which significantly exceeding the antioxidant content. In a recent study by [33], the antioxidant values were reported in *P. oleracea* (2378.2 µmol TE·100 g^−1^ dm) and *P. ruderale* (6355.0 µmol TE·100 g^−1^ dm), as raw materials obtained by the same method, with a difference in the preparation of samples and reagents. The antioxidant levels found in *P. ruderale* exceed those found in our study and do not reach those of *P*. *oleracea*, which may be due to the fact that the results are expressed as dry material. The antioxidant levels suggest *P. oleracea* as a very promising product. Similarly, the antioxidant content of *P. ruderale* leaves stands out, whose results, expressed on a fresh weight basis (fw), were not able to be contrasted due to the lack of publications. The review article by Marquez et al. [50] corroborates that most articles on *P. ruderale* have been published in the areas of biological and pharmacological science in the last 39 years.

It can be highlighted that the results of this study distinguish the species *P. oleracea* and *P. ruderale*, characteristic of the Mediterranean spring-summer season, as powerful sources of antioxidants, which, in general, reverse the damage caused by oxidative stress and prevent the appearance of pathologies that involve it. According to the database [47], vegetables with similar values in the content of total antioxidants are fresh arugula, with 5998 µmol TE·100 g^−1^ fw, and fresh spinach, with 5432 µmol TE·100 g^−1^ fw. Much higher is fresh parsley, with 28,865 µmol TE·100 g^−1^ fw. The results were determined by the ORAC test.

Total phenolic content had been reported to be associated with total antioxidant activity in both studied species. The higher recorded content was for wild samples. The research in [51] established the variation of total phenols of the Malaysian cultivars of *P. oleracea* in the range of 127 ± 13 to 478 ± 45 (mg GAE·100 g^−1^ fw). The values found in our study for wild *P. oleracea* are close to the levels reported but, on the contrary, cultivated species presented lower values. The correlation analysis between components confirms that the richness of antioxidants is strongly related to the presence of total polyphenols in *P. oleracea*, although its wild species far exceeds that cultivated by organic techniques. Additionally, for *P. ruderale* there is no strong correlation between antioxidant and phenolic compounds, suggesting the presence of other antioxidant components such as anthocyanins, vitamins, or carotenoids, which requires further investigation.

Phenolic compounds are present in plants and take part in their defense, while for humans they have many benefits [52]. In the current study, the content of phenolic compounds showed significant variability between growing systems and species. A study conducted by [53] asserted that phenolic content fluctuated between different growing conditions. As indicated by the authors of [54], the amount of hydroxycinnamic acids depend on the vegetative progress of the plants, and its highest content is found in developing leaves that have greater metabolic activity. This means that in *P. oleracea,* in our study, gallic acid, rutin, and luteolin were not detected, and apigenin was only detected in organic cultivation conditions, probably collected in an optimal state of development, possibly due to genetic factors, but also due to a very mature state of the plant. In contrast, in other work, quercetin and kaempferol were not found in Turkish *P. oleracea* [55], so the plant’s age may be important for the synthesis of these compounds. Ferulic and rosmarinic acid were not found in our samples of Malaysian *P. oleracea*, in contrast to [51]. Chlorogenic acid was the dominant hyrdoxycinnamic acid in *P. ruderale*. The polyphenolic profile study carried out is in line with the correlations made previously with the content of total phenolic content (TPP). In *P. oleracea,* antioxidants and phenols have a strong relationship despite the absence of some polyphenolic compounds in its profile, especially flavonoids. In contrast, *P. ruderale,* whose polyphenolic profile showed a greater amount of compounds, especially flavonoids, provided moderate antioxidant power. This indicates that this species has other bioactive compounds such as antioxidants that could be the subject of future research.

In the research conducted by Udeagha et al. [56], they assert that chlorophyll is involved in the synthesis of cell growth molecules, making it a key indicator of the physical state of the plant, reflecting its photosynthetic capacity, productivity, and stress level, among other aspects. Considering that, due to the amount of total chlorophyll, spinach is the raw material par excellence for the industrial extraction of chlorophyll, with an average of 16 μg·g^−1^. It was observed that the study species showed a moderate level of chlorophyll in cultivated *P. ruderale,* and low levels in both *P. oleracea* growing systems. Wild *P. ruderale* showed a considerable level of total chlorophyll. On the other hand, chlorophyll values as technical-scientific information allows the identification of superior genotypes in the genetic improvement process to improve productivity. In addition, according to [57], chlorophyll is a bioactive component that reduces high levels of cholesterol and triglycerides and can improve health in a balanced diet with the intake of green leafy vegetables such as the undervalued species of this study. These species can also add color to gastronomic dishes due to their organoleptic qualities. Furthermore, natural chlorophylls have an effect on inflammation and wound healing, and prevent lipid peroxidation [58].

The nitrate content determined in this study was shown to be higher in wild species than in their cultivated counterparts. According to [59], regarding the classification of vegetables in the content of nitrates, *P. ruderale* belongs to the medium category, with a nitrate range of 500–1000 mg NO_3_^-^·kg^−1^ fw, and *P. oleracea* to the low category (200–500 mg NO_3_^−^·kg^−1^ fw). Nitrates are naturally occurring compounds in the environment due to the nitrogen cycle, but they can be altered by various agricultural practices. Nitrate quantification is used to diagnose the nutritional status of plants, particularly crop plants, as they accumulate nitrates in their chloroplasts. Nitrate itself is relatively toxic. Its toxicity is determined by its conversion to nitrite, which can be influenced by agronomic factors, and sometimes by processing or cooking techniques [60]. To comply with the hygienic-sanitary characteristics, the WHO/FAO acceptable daily intake is 3.7 mg NO_3_^−^·kg^−1^ of body weight, expressed as nitrate ions. However, some studies report that nitrate or diets moderately rich in this chemical component can induce a reduction in pressure [61,62]. Principally, nitrate concentration in vegetables varies according to climatic conditions and agronomic crop management, as well as post-harvest storage conditions [63,64]. Nitrates accumulate in plant vacuoles depending on the fraction absorbed from the soil. Subsequently, this compound is reduced to ammoniacal and amino forms for the formation of protein. The reduction process is conditioned by two enzymes that require metal cofactors such as iron and molybdenum. It is possible that organic cultivation conditions favor a balance of factors that cause a reduction, decreasing the concentration of nitrates in the edible leaves [65]. There is a clear need for future studies in antinutritional compounds to ensure the complete safety of these undervalued plants.

The pH values of the aerial parts of wild species were higher than in their cultivated relatives. At the same time, the highest total acidity was found in the cultivated species. Both parameters indicate the presence of organic acids in the samples. The influence of pH on the assimilation of macro and micronutrients may be the most important factor, and at the same time, the pH and the total titratable acidity influence its organoleptic properties, giving a specific acid taste to the plants. Variations in these parameters can be attributed to environmental and cultivation conditions. In this sense, wild growing conditions can generate more palatable species from an organoleptic point of view by balancing their acids.

The statistical treatments applied indicated low variability in most of the nutritional values in *P. ruderale* (wild and organically cultivated). The small number of samples influenced the width of confidence intervals for bioactive compounds. The *p*-value corroborated significant differences in the parameters studied according to the growing conditions. The study of correlations between nutritional and bioactive compounds as quality parameters showed different degrees of correlation between them. In *P. ruderale,* highly significant negative relationships prevailed, while in *P. oleracea,* the significant relationships were mostly positive. The absence of strong relationships between Zn (*P. oleracea*), K, and Na (*P. ruderale*) may be due to species-specific biosynthesis/transport processes that are influenced by geoclimatic conditions during growth depending on the harvest period, as suggested by [58,66]. The complete relationship between Na and P (*P. oleracea*) can be explained by the fact that the studied species are halophyte plants [67]. The correlations of different degrees found between compounds in both plants were weak, especially for total chlorophyll (*P. oleracea*). This might be due to common factors in the secondary metabolite’s synthesis pathway, and in the case of chlorophyll, there is a dependence on foliar anatomy [68].

The analysis of the volatiles of fresh leaves showed specificity, and each species could be differentiated from others based on its aroma profile, which is strongly influenced by volatile components. The volatile analysis showed that monoterpenoids and unsaturated hydrocarbons were the most common volatile families in *P. ruderale*, and benzenoids were the most common in *P. oleracea*. Terpenoid compounds such as aldehydes and terpenes possibly contribute to antioxidant properties [69]. Under abiotic and biotic stress conditions, the production of benzenoid family metabolites is intensified; the same influence is exerted by heat stress, which intensifies the biosynthesis of benzenoids [70]. Fatty alcohols were found to be distinctive in *P. oleracea*. Most alcohols displayed an unmistakable fragrance of green scents and contributed to the perception of grassiness [71]. Medium-chain aldehydes were found to be homogeneous in the cultivated and wild species of both plants. The components of this family, especially (E)-2-hexenal, are known as green leaf aldehydes, which provide a characteristic scent. In addition, this constituent may be involved in the abiotic stress response [72]. Sesquiterpenoids predominated in wild *P. ruderale* and ketones predominated in cultivated *P. oleracea*. Sesquiterpenoids may play a significant role in human health due to their potential in preventing cardiovascular disease and cancer [73].

Among the minority families, organoheterocyclic and organooxygen compounds stood out in *P. oleracea*, as well as the family of alcohols, whose compounds are identified as major predictors for the freshness index [74]. The pyrazine family predominated in *P. oleracea* under both growing conditions. Pyrazines are considered a key family in intensity of smell in nature [75]. The volatiles analyzed showed a difference in the relative abundance of the majority and minority chemical groups found in this study. Aroma properties depend on the combination of volatiles and their concentrations, and can be an index of quality.

## 4. Materials and Methods

### 4.1. Plants Materials

*P. oleracea* (purslane) is an annual herb that has become naturalized throughout the world; it is considered a weed in some regions. The stems, leaves, and flowers are edible. It has a slightly acidic and salty taste and can be eaten fresh in salad or cooked as a leafy vegetable [76]. *P. ruderale* is an annual herb native to Central and South America, but is adapted to different edaphoclimatic conditions. It is known as ‘‘rupay wachi’’ and “quirquiña”, among other common names [77]. This species is a wild herb with a strong and distinctive flavor; the leaves and tender stems are commonly used for salads and spicy sauces [78]. In addition, both plants have some phytopharmaceutical properties, provided by their biologically active compounds.

The species were collected between June–July 2020 from two environments along the Valencian coast (Spain): (1) organic farming and (2) wild conditions. Organic cultivation methods were carried out on the Unió de Llauradors i Ramaders farm, which was organic certified 18 years ago. The area is located within 39°45′13′′ North and 0°12′21′′ West, with SCI code ES0000148 [79]. The edible aerial parts such as leaves and small tender stems were separated and used for extractions and analytical quantification of bioactive compounds; methanolic extracts were prepared to determine total antioxidants, and acetone extracts to determine the chlorophyll content, as well as an aqueous extract for total polyphenols and other chemical constituents such as nitrates, pH, and total acidity. The rest of the determinations were performed with plant matter dried to a constant weight in an autoclave (J.P. Selecta) at 70 ± 0.1 °C. The dry vegetable fraction was ground with a grinding mill (Retsch KG-5657 Haan, Remscheid, Germany) to obtain a fine powder (250 µm) and stored in high-density polyethylene bottles under refrigeration conditions at 4 °C for subsequent analysis (nutritional and mineral compositions).

### 4.2. Chemical Reagents

Solutions of 80% (*v*/*v*) methanol and 80% (*v*/*v*) acetone were prepared from analytical grade reagents. The chemicals were sodium carbonate, citric acid, boric acid, sulfuric acid, hydrochloric acid, phosphoric acid, lanthanum (III) chloride, and sodium hydroxide (Scharlau). Trolox (6-hydroxy-2,5,7,8, -tetramethyl-chroman-2-carboxylic acid), 2,2′-azobis-2-methyl-propanimidamide, 1,1-diphenyl-2-picrylhydrazyl (DPPH), Folin–Ciocalteu reagent (FCR), and gallic acid were purchased from Sigma-Aldrich Co (Taufkirchen, Germany). Water was prepared using a Water Still Aquatron A4000 distiller. Standard references for phenolic compounds were from Sigma-Aldrich Co., St. Louis, MI, USA.

### 4.3. Nutritional Characteristics

Prior to sample analysis, all analytical methods were optimized and fine-tuned for the specific analysis of this type of matrix. Three replicates were performed for each analysis. 

#### 4.3.1. Proximate Composition

Analysis was carried out by official methods [80]: moisture (AOAC 984.25), crude protein (AOAC 984.13), fat (AOAC 983.23), crude fiber (AOAC 991.43), and ash (AOAC 923.03). The carbohydrate content was calculated by difference. The results were expressed in g·100 g^−^^1^ fresh weight (fw).

#### 4.3.2. Mineral Composition

The samples were subjected to digestion in accordance with method AOAC 985.35. The samples were calcined in a Carbolite CWF 1100 chamber furnace at 550 °C, and the ashes were dissolved with concentrated HCl until a 2% HCl solution was obtained. Calibration curves were established using working standards for each element. The analytical curves were obtained with a linear response for the selected concentration ranges. Mineral analysis was performed by atomic absorption spectroscopy (AAS) in a THERMO elemental AA series Spectrometer (spectrophotometer), v.11.03 software, and hollow cathode lamps for each element, except for phosphorus, which was analyzed by colorimetry [80].

### 4.4. Non-Nutritional Compounds

**Figure 3:**
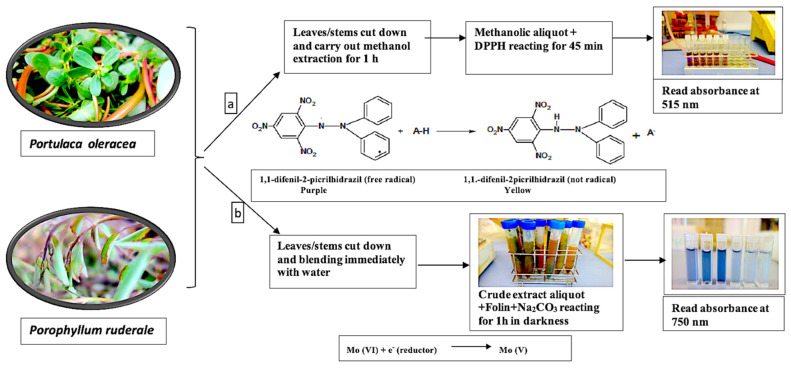
Reaction process in the quantification of bioactive constituents: (**a**) total antioxidants with the reaction of the DPPH radical; (**b**) total polyphenols with electron transfer reaction with the FCR.

To determine total antioxidants, the 0.8 g of fresh leaves and small tender stems were mixing in 5 mL of methanol solution (80% *v*/*v*) and stirred for 1 h at room temperature by an SO1 orbital shaker (Bibby Stuart Scientific, Staffordshire, UK). Then, reagent was added to measure the effects of the extract on the DPPH radical. The calibration curve was obtained with the Trolox standard. The summary process and resulting reaction are shown in Figure 3a.

To determine the total phenolic content, the aerial parts of fresh plants were crushed with water at a ratio of 2:1 (solvent: plant) and immediately reacted with FCR. The calibration curve was obtained with the gallic acid standard. The summary of the process and the reaction are show in Figure 3b.

Prior to the analysis of the samples, all analytical methods were optimized and tuned for the specific analysis of this type of matrix. Table 5 shows the values of linearity, calibration curve, linear range, and retention time (Tr) for the main non-nutritional compounds.

#### 4.4.1. Total Antioxidants 

The 25 ppm DPPH solution was prepared in 80% methanol (*v*/*v*) and 3.9 mL of this solution was mixed with 0.1 mL of methanolic extract aliquot to initiate the reaction. After incubation for 45 min at 23 °C, the progress of the reaction was monitored at 515 nm [81]. Trolox was used as the standard for analysis. The results were expressed as µmol Trolox equivalents per 100 g of fresh weight (µmol TE·100 g^−1^ fw).

#### 4.4.2. Total Phenolic Content

The optimized Folin–Ciocalteu method [82] consisted of preparing a series of spectrophotometric cuvettes with 50 µL of aqueous extract aliquot. To this was added 0.5 mL of the FCR (previously diluted with 1:10 *v*/*v* water). Before 8 min, 0.5 mL of 6% (*w*/*v*) Na_2_CO_3_ solution was added. After the reaction, the absorbance at 750 nm was measured by spectrophotometer (JENWAY 6715/UV-Vis). The results are expressed as mg gallic acid equivalents per 100 g of fresh weight (mg GAE·100 g^−1^ fw).

#### 4.4.3. Polyphenols Profile by HPLC 

The sample (2 g dry leaves) was subjected to direct solvent extraction (15 mL) of 75% ethanol (*v*/*v*) with agitation on an orbital shaker for 2 h, followed by centrifugation and filtration. The extracted phenolic acid and flavonoids were individually quantified and separated through an HPLC system (HPLC, Agilent 1220 Infinity LC) equipped with a UV detector. The analytical column was a Brisa C18 (150 × 4.6 mm, particle size = 3 µm, Teknokroma). The mobile phase contained water with 0.1% formic acid (A) and methanol with 0.1% formic acid. The temperature was set at 30 °C and the sample injection volume was 10 µL. The chromatography was performed under the following conditions:

Hydroxycinnamic acids: flow rate 1.0 mL/min flow rate; gradient program: 0–8 min 7% B, 8–13 min 30% B, 13–48 min 66% B, 48–50 min 66% B, 50–56 min 100% B, 56–65 min 7% B. Polyphenols were detected by monitoring the absorbance at 280/320 nm [83].

Flavonoids: flow rate 0.8 mL/min flow rate; gradient program: 0–10 min 40% B, 10–15 min 100% B, 15–20 min 40% B, 20–25 min 40% B at absorbance 360 nm [84]. The results were expressed as μg·g^−1^ fresh weight.

#### 4.4.4. Chlorophylls: a, b, Total

The extraction of chlorophylls was carried out by adapting the method proposed in [85]. The ground aerial parts were suspended in pigment extraction solvent (80% acetone *v*/*v*), filtered through filter paper to avoid turbidity, and brought up to a volume of 50 mL with the same extraction solvent. Solutions were measured spectrophotometrically at 645 nm, 653 nm, and 663 nm. The results were expressed as μg·g^−1^ fw on a per sample basis.

### 4.5. Other Chemicals: Nitrates, Ph and Total Acidity

Aqueous extracts from fresh aerial parts were prepared in a 1:2 (*w*/*v*) ratio at a temperature lower than 30 °C by mechanical grinding. Nitrates (mg NO_3_^-^·kg^−1^ fw) and pH were measured directly by pH and ION-Meter GLP 22+ (CRISON) equipment and with the respective electrodes, after calibrating each electrode. The total acidity was determined potentiometrically with 0.05 N NaOH solution, and the results are expressed as a percentage of citric acid.

### 4.6. Volatiles Profile Analysis

Dynamic headspace sampling was used to analyze the volatiles present in fresh leaf and stem samples. Extraction of volatile compounds was carried out using the HS-SPME technique according to [86]. The analysis was performed by a 6890 N gas chromatography and mass spectrometry (GC-MS) networked to a 5973 inert mass selective detector (Agilent Technologies). The analytical conditions were as follows: stationary phase HP-5MS J&W silica capillary column (30 m × 0.251 mm i.d. × 0.25 µm thickness film; 5% phenyl-95% methylpolysiloxane); helium carrier gas at a constant flow of 1 mL min^−1^; transfer line maintained at 220 °C. Initial temperature (40 °C) for 1 min, Ramp 1 from 5 °C min^−^^1^ up to 200 °C for 1 min, and Ramp 2 from 15 °C min^−^^1^ up to 250 °C for 3 min. The electron impact mode with ionization energy of 70 eV (source temperature 225 °C) was used for detection by the mass spectrometer, and the acquisition was performed in scan mode (mass range *m*/*z* 35–350 amu).

### 4.7. Statistical Analysis

Datasets from wild and cultivated species were processed using Statgraphics Plus version 5.1 (Manugistics Inc., Rockville, MD, USA) for means, standard errors, and correlations. The analysis of variance (multivariate ANOVA; at a significant level of *p* < 0.05) was performed according to a completely randomized design with three replicates. Differences between groups were identified with multiple comparisons of means (Tukey contrast), and the bivariate statistical method was applied to determine the relationship between the various qualitative characteristics of the plants studied. The independent variables defined the qualitative parameters, and the dependent variables were the species and the growing conditions. Pearson linear correlation coefficients (r) between traits were calculated from regression analyses between pairs of traits. The confidence limits used in this study were based on 95% (*p* < 0.05), and in polyphenol individuals, on 99% (*p* < 0.01).

## 5. Conclusions

The results of this study provide a basis for the characterization of nutritional, organoleptic, and non-nutritional compounds of *P. ruderale* and *P. oleracea*. These undervalued plants have significant crude fiber, carbohydrate, mineral, and chlorophyll content, and are a good source of antioxidants and phenolic compounds. Studies of polyphenolic and volatile profiles show that both species possess bioactive compounds with functional properties. These preliminary data reveal that these plants are a promising source of new natural antioxidants, as well as possible material for new improved varieties. Their production potential can boost local economies and ensure ecological security, as the species studied grow in diverse habitat conditions. In addition, their nutritional quality and promising amount of bioactive components will greatly contribute to knowledge about these undervalued plants that, due to their nutrient-dense characteristics and low energy content, could be part of health diets.

## Figures and Tables

**Table 1:** Nutritional and bioactive compounds: mean value ± standard error for each parameter analyzed; coefficient of variability (CV) and probability (*p*-value) for the significance of differences between the environmental growing conditions of *P. ruderale* and *P. oleracea*.

		*P. ruderale*					*P. oleracea*				
		Wild	CV (%)	Cultivated	CV (%)	*p*-Value	Wild	CV (%)	Cultivated	CV (%)	*p*-Value
Nutritional value (g 100 g^−1^ fw)	Moisture	84.70 ^a^ ± 0.43	0.51	76.64 ^b^ ± 0.02	0.03	0.0000	88.39 ^a^ ± 0.24	0.27	83.12 ^b^ ± 1.09	1.31	0.0014
	Dry matter	15.30 ^b^ ± 0.61	3.99	23.36 ^a^ ± 0.17	0.73	0.0000	11.61 ^b^ ± 0.37	3.19	16.88 ^a^ ± 0.85	5.04	0.0014
	Ash	1.49 ^b^ ± 0.02	0.28	2.33 ^a^ ± 0.02	0.15	0.0020	2.62 ^b^ ± 0.02	0.76	3.39 ^a^ ± 0.06	1.77	0.0084
	Crude proteins	1.89 ^a^ ± 0.00	0.05	1.19 ^b^ ± 0.01	0.84	0.0002	1.56 ± 0.00	0.06	1.49 ± 0.00	0.07	0.1988
	Fat	0.66 ^a^ ± 0.00	0.15	0.41 ^b^ ± 0.01	2.44	0.0156	0.32 ^b^ ± 0.00	0.31	0.99 ^a^ ± 0.00	0.10	0.0000
	Crude fiber	5.50 ± 1.88	34.18	3.57 ± 0.66	18.64	0.1003	2.39 ± 0.01	0.42	2.60 ± 0.89	34.23	0.7178
	Carbohyrates	6.80 ^b^ ± 3.49	51.32	17.55 ^a^ ± 0.63	3.59	0.0008	4.72 ^b^ ± 0.21	4.45	8.41 ^a^ ± 0.90	10.22	0.0183
	Energy value (kcal 100 g^−1^)	40.70 ± 1.16	2.85	78.65 ± 0.22	0.28	-	28.00 ± 0.07	0.25	48.51 ± 0.30	0.62	-
Minerals (mg 100 g^−1^ fw)	Calcium	439.29 ^b^ ± 119.86	27.29	687.49 ^a^ ± 19.22	2.80	0.0240	186.67 ^a^ ± 28.36	15.19	110.59 ^b^ ± 16.02	14.49	0.0005
	Magnesium	131.15 ^b^ ± 9.22	7.03	185.54 ^a^ ± 21.10	11.37	0.0150	165.33 ^a^ ± 9.50	5.75	91.68 ^b^ ± 18.91	20.63	0.0000
	Potassium	515.28 ± 49.22	9.55	477.75 ± 40.99	8.58	0.3676	776.67 ^a^ ± 171.50	22.08	271.91 ^b^ ± 34.37	12.64	0.0000
	Phosphorus	56.48 ^b^ ± 3.31	5.86	84.57 ^a^ ± 3.96	4.68	0.0007	33.67 ^b^ ± 0.93	2.76	58.73 ^a^ ± 7.56	12.87	0.0000
	Sodium	7.19 ± 1.21	16.83	8.21 ± 1.60	19.49	0.4337	16.60 ^a^ ± 0.03	0.18	0.81 ^b^ ± 0.06	7.41	0.0000
	Iron	1.80 ± 0.12	6.67	1.70 ± 0.17	10.00	0.4495	1.80 ± 0.18	10.00	1.35 ± 0.18	13.33	0.1960
	Copper	0.17 ^b^ ± 0.01	5.88	0.37 ^a^ ± 0.01	2.70	0.0000	0.14 ^b^ ± 0.01	7.14	0.36 ^a^ ± 0.05	13.89	0.0000
	Zinc	0.51 ^a^ ± 0.04	7.84	0.39 ^b^ ± 0.03	7.69	0.0157	0.99 ^b^ ± 0.06	6.06	1.08 ^a^ ± 0.14	12.96	0.0011
Bioactive compounds	TAO (µmol TE·100 g^−1^ fw)	4645.53 ^a^ ± 36.2	0.78	4392.16 ^b^ ± 27.0	0.62	0.0006	7315.0 ^a^ ± 386.30	5.28	4609.98 ^b^ ± 168.3	3.65	0.0004
	TPP (mg GAE·100 g^−1^ fw)	391.18 ± 141.50	36.17	316.20 ± 28.90	9.14	0.4152	318.93 ^a^ ± 40.20	12.60	99.09 ^b^ ± 35.50	35.83	0.0021
	Chl a (μg·g^−1^fw)	10.45 ^a^ ± 0.52	4.98	5.55 ^b^ ± 0.53	9.55	0.0003	3.35 ± 0.11	3.28	3.37 ^b^ ± 0.33	9.79	0.9434
	Chl b (μg·g^−1^fw)	3.55 ^a^ ± 0.28	7.89	1.70 ^b^ ± 0.15	8.82	0.0006	1.85 ^a^ ± 0.15	8.11	1.30 ^b^ ± 0.13	10.00	0.0002
	Total Chl (μg·g^−1^fw)	14.00 ^a^ ± 0.78	5.57	7.25 ^b^ ± 0.67	9.24	0.0003	5.20 ± 0.26	5.00	4.66 ± 0.44	9.44	0.1450

Note: TAO: total antioxidants (TE = Trolox equivalent); TPP: total phenols (GAE = gallic acid equivalent); Chl: chlorophyll. Means followed by superscript letters (a–d) are significantly different (*p* < 0.05).

**Table 2:** Individual hydroxycinnamic acid and flavonoid content of *P. ruderale* and *P. oleracea* species.

		*P. ruderale*				*P. oleracea*			
		Wild	Cultivated	*p*-Value	S	Wild	Cultivated	*p*-Value	S
Hydroxycinnamic acids (μg·g^−1^ fw)	Gallic acid	1.13 ± 0.07	0.41 ± 0.07	0.0002	**	nd	nd	-	-
	Chlorogenic acid	798.45 ± 36.52	780.08 ± 0.85	0.4329	ns	6.75 ± 1.09	11.38 ± 2.64	0.0399	*
	Caffeic acid	3.93 ± 0.16	1.67 ± 0.03	0.0000	**	5.72 ± 0.29	16.25 ± 0.82	0.0000	**
	*p*-Coumaric acid	54.8 ± 2.26	175.21 ± 1.28	0.0000	**	4.21 ± 0.17	17.99 ± 1.19	0.0000	**
Flavonoids (μg·g^−1^ fw)	Myricetin	nd	1.54 ± 0.10	-	-	0.95 ± 0.04	10.26 ± 0.62	0.0000	**
	Rutin	14.73 ± 0.70	15.43 ± 0.46	0.2242	ns	nd	nd	-	-
	Quercetin	40.42 ± 2.44	49.95 ± 0.86	0.0031	*	0.21 ± 0.03	0.47 ± 0.01	0.0002	**
	Luteolin	3.98 ± 0.22	7.3 ± 0.13	0.0000	**	nd	nd	-	-
	Kaempferol	5.84 ± 0.36	13.76 ± 0.61	0.0000	**	0.09 ± 0.01	0.30 ± 0.04	0.0006	**
	Apigenin	2.48 ± 0.36	3.59 ± 0.09	0.0068	*	nd	0.96 ± 0.06	-	-

Note: All data are expressed as means ± standard error, *n* = 3; ns, * and, ** indicate that the F test is not significant or significant at *p* < 0.05 and *p* < 0.01, respectively. Wild and organic cultivated growth conditions were compared by *t*-test; nd: not detected; S: significance.

**Table 3:** Pearson’s correlation coefficient among the nutritional, mineral, and bioactive compounds of edible parts of *P. ruderale*.

	CP	FT	CF	CH	Ca	Mg	K	P	Na	Fe	Cu	Zn	TAO	TPP	TCh
FT	0.879 *	1													
CF	0.685	0.798	1												
CH	−0.956 *	−0.908 *	−0.856 *	1											
Ca	−0.854 *	−0.740	−0.792	0.914 *	1										
Mg	−0.949 *	−0.836 *	−0.634	0.871 *	0.771	1									
K	0.465	0.298	0.625	−0.541	−0.439	−0.481	1								
P	−0.947 *	−0.938 *	−0.832 *	0.989 *	0.868 *	0.840 *	−0.461	1							
Na	−0.410	−0.054	−0.036	0.317	0.17	0.313	−0.615	0.298	1						
Fe	0.463	0.209	0.139	−0.335	−0.109	−0.540	0.721	−0.296	−0.814 *	1					
Cu	−0.983 *	−0.917 *	−0.715	0.967 *	0.834 *	0.888 *	−0.415	0.980 *	0.385	−0.386	1				
Zn	0.902 *	0.656	0.599	−0.870 *	−0.940 *	−0.837 *	0.446	−0.817 *	−0.395	0.321	−0.848 *	1			
TAO	0.950 *	0.928 *	0.812 *	−0.983 *	−0.835 *	−0.842 *	0.499	−0.996 *	−0.368	0.368	−0.983 *	0.802	1		
TPP	0.430	0.438	−0.137	−0.244	0.045	−0.391	−0.210	−0.342	−0.326	0.373	−0.475	0.124	0.386	1	
TCh	0.990 *	0.836 *	0.606	−0.929 *	−0.834 *	−0.908 *	0.402	−0.928 *	−0.464	0.446	−0.980 *	0.908 *	0.934 *	0.484	1

* indicates significant difference at *p* < 0.05. CP = crude protein; CF = crude fiber; CH = carbohydrates; TAO = total antioxidants; TPP = total polyphenols; TCh = total chlorophyll.

**Table 4:** Pearson’s correlation coefficient among the nutritional, mineral, and bioactive compounds of edible parts of *P. oleracea*.

	CP	FT	CF	CH	Ca	Mg	K	P	Na	Fe	Cu	Zn	TAO	TPP	TCh
FT	−0.597	1													
CF	−0.739	0.174	1												
CH	−0.517	0.880 *	−0.048	1											
Ca	0.403	−0.884 *	−0.034	−0.808	1										
Mg	0.438	−0.942 *	0.001	−0.930 *	0.956 *	1									
K	0.419	−0.918 *	−0.166	−0.787	0.954 *	0.951 *	1								
P	−0.704	0.947 *	0.430	0.713	−0.764	−0.804	−0.852*	1							
Na	0.611	−0.999 *	−0.185	−0.889 *	0.895 *	0.949 *	0.924 *	−0.943 *	1						
Fe	0.359	−0.809	−0.100	−0.841 *	0.879 *	0.929*	0.927*	−0.684	0.823*	1					
Cu	−0.663	0.970 *	0.343	0.760	−0.793	−0.842 *	−0.870 *	0.995 *	−0.965 *	−0.709	1				
Zn	−0.803	0.467	0.739	0.143	−0.208	−0.178	−0.267	0.690	−0.460	−0.014	0.635	1			
TAO	0.634	−0.979 *	−0.262	−0.831 *	0.933 *	0.937 *	0.946 *	−0.941 *	0.983 *	0.823 *	−0.953 *	−0.500	1		
TPP	0.774	−0.963 *	−0.338	−0.853 *	0.771	0.850 *	0.810	−0.956 *	0.964 *	0.705	−0.965 *	−0.624	0.939 *	1	
TCh	−0.664	0.001	0.589	0.164	−0.071	−0.040	0.007	0.033	−0.036	−0.140	−0.0133	0.253	−0.106	−0.167	1

* indicates significant difference at *p* < 0.05. CP = crude protein; CF = crude fiber; CH = carbohydrates; TAO= total antioxidants; TPP= total polyphenols; TCh = total chlorophyll.

**Table 5:** Linearity, calibration curve, linear range, and retention time for main non-nutritional compounds.

Compound	Calibration Curve	Linearity	Linear Range	Tr (min)
AOT	y= −0.1347x + 1.0678	R^2^ = 0.9783	0.3–1.3 μM	-
TPP	y= 0.0018x + 0.0182	R^2^ = 0.9898	0–400 ppm	-
Gallic acid	y = 25.36x + 21.562	R^2^ = 0.9992	2–350 ppm	6.39
Chlorogenic acid	y = 25.567x + 32.541	R^2^ = 0.9998	2–300 ppm	16.63
Caffeic acid	y = 45.356x + 37.156	R^2^ = 0.9990	1.72–220 ppm	17.74
*p*-Coumaric acid	y = 60.223x + 228.38	R^2^ = 0.9992	3–440 ppm	21.26
Myricetin	y = 41.599x + 10.346	R^2^ = 0.9987	0.75–48 ppm	6.26
Ruine	y = 20.071x + 5.5868	R^2^ = 0.9984	1.4–94 ppm	7.02
Quercetin	y = 44.696x + 26.656	R^2^ = 0.9984	1.6–110 ppm	8.16
Luteolin	y = 21.996x – 10.039	R^2^ = 0.9997	1.5–98 ppm	8.59
Kaempherol	y = 51.019x + 23.177	R^2^ = 0.9986	1.5–97 ppm	9.13
Apigenin	y = 31.051x + 18.277	R^2^ = 0.9985	1.5–95 ppm	9.38

## Data Availability

The individual data presented in this study are available on request from the corresponding author.
